# Best Practice Guidelines for Pre-Launch Characterization and Calibration of Instruments for Passive Optical Remote Sensing^1^

**DOI:** 10.6028/jres.116.009

**Published:** 2011-04-01

**Authors:** R. U. Datla, J. P. Rice, K. R. Lykke, B. C. Johnson, J. J. Butler, X. Xiong

**Affiliations:** National Institute of Standards and Technology, Gaithersburg, MD 20899-0001; NASA Goddard Space Flight Center

**Keywords:** best practice guidelines, radiometric calibrations, remote sensing, SI traceability

## Abstract

The pre-launch characterization and calibration of remote sensing instruments should be planned and carried out in conjunction with their design and development to meet the mission requirements. The onboard calibrators such as blackbodies and the sensors such as spectral radiometers should be characterized and calibrated using SI traceable standards. In the case of earth remote sensing, this allows inter-comparison and intercalibration of different sensors in space to create global time series of climate records of high accuracy where some inevitable data gaps can be easily bridged. The recommended best practice guidelines for this pre-launch effort is presented based on experience gained at National Institute of Standards and Technology (NIST), National Aeronautics and Space Administration (NASA) and National Oceanic and Atmospheric Administration (NOAA) programs over the past two decades. The currently available radiometric standards and calibration facilities at NIST serving the remote sensing community are described. Examples of best practice calibrations and intercomparisons to build SI (international System of Units) traceable uncertainty budget in the instrumentation used for preflight satellite sensor calibration and validation are presented.

## 1. Introduction

Satellite remote sensing provides continuous coverage and has the potential to allow observation of climate variables through long time periods. Climate modelers require continuous data over long time periods to test their models and predict global climate variability. However, the data has to be accurate and the measurement uncertainties well understood to be of value to the modelers. Two workshops were held to identify the accuracy requirements for radiometric measurements and identify ways to achieve those goals [1, 2]. Table 1 shows the required accuracies and stabilities for climate variable data sets and Table 2 shows the corresponding radiometric accuracies and stabilities of satellite instruments to meet those requirements, based upon the workshops [1]. The requirements are very demanding and the golden rule for achieving the needed accuracy is to make measurements traceable to international standards (SI) [2]. The result of a measurement can be traceable to international standards through an unbroken chain of comparisons all having stated uncertainties [3]. The recommended practice to evaluate and document the stated uncertainties is to follow the International Organization for Standardization (ISO) Guide to expression of uncertainty in measurement [4]. This process allows uniformity and intercomparability of measurements on different satellite platforms in space taken simultaneously as well as at different times potentially spanning decades as needed for climate observations. In Sec. 2, we will discuss SI traceability and best practice for pre-launch characterization and calibration of sensors for achieving the measurement accuracy goals on-orbit. In Sec. 4, the absolute standards for radio-metric scales at NIST are presented. In Sec. 5, the transfer radiometers at NIST and those built by NIST for NASA for SI traceability are discussed. In Sec. 6, illustrations of best practice for calibrations and intercomparisons to build SI traceable uncertainty budgets for instrumentation used for sensor calibrations for customers are discussed. Concluding remarks are given in Sec. 7.

## 2. SI Traceability and Best Practice

A question often raised is, what is the difference between having SI traceability as a requirement versus not having that stated in the requirements? The difference is that such a requirement specifically mandates that the characterizations and calibrations are to be performed against standards traceable to the SI. Also, the uncertainties are to be carefully evaluated, tabulated component by component, and the total uncertainty budget is to be made transparent for peer review and independent critical analysis. There are two kinds of uncertainties to be evaluated according to the ISO Guide [4] called Type A and Type B. Type A uncertainties are basically the random type and represent the uncertainty in the repeatability of measurements. In general, because of good environmental control on the instrumentation and computer acquisition and analysis of the data at a fast rate, the random uncertainties can be made very small in the pre-launch phase. However, these uncertainties have to be re-characterized post-launch and periodically re-assessed on orbit using space view of the sensor. While on orbit there may be good repeatability within a short measurement time interval, in a long time series of measurements the sensor may have a drift due to its degradation in the space environment. This is a systematic effect which could be corrected if measured or scientifically estimated. The systematic effect or its correction will have an uncertainty that must be estimated based on the ISO guide. The systematic uncertainties evaluated in the characterization of various parts of the sensor system are called Type B uncertainties; and they are also to be evaluated in the pre-launch and post-launch mission phases. The square root of the sum of the squares (RSS) of these two types of uncertainties gives the combined standard uncertainty, *u*_c_ and an expanded uncertainty *U_p_* = *k_p_ u*_c_ where *k_p_* is called the coverage factor. For a normal distribution, the level of confidence *p* for *k_p_* = 1 corresponds to 68.3 %. For example, in Table 1, the accuracy and stability requirements are quoted for *k_p_* = 1. (In current usage of this coverage factor *k_p_* is abbreviated as *k*). In the remote sensing terminology the ability of the sensor to maintain its repeatability over a period of time is called the stability of the sensor. Stability is measured by the maximum excursion (drift) of the short-term average measured value of a variable after appropriate corrections through on orbit calibrations under identical conditions over a decade. The accuracy is a measure of the closeness of the result of measurement and the true value. It is measured as the standard uncertainty of the combined result of all measurements. Both these quantities are prone to have Type A and Type B uncertainties. In practice, based on the standard uncertainties in Table 1, the expanded uncertainties are to be used with appropriate coverage factors to define the interval for expressing the level of confidence for the measured value to lie [1, 2].

### 2.1 Best Practice for Characterization/Calibration of Satellite Optical Instruments—3 Step Process

**Fig. 1:**
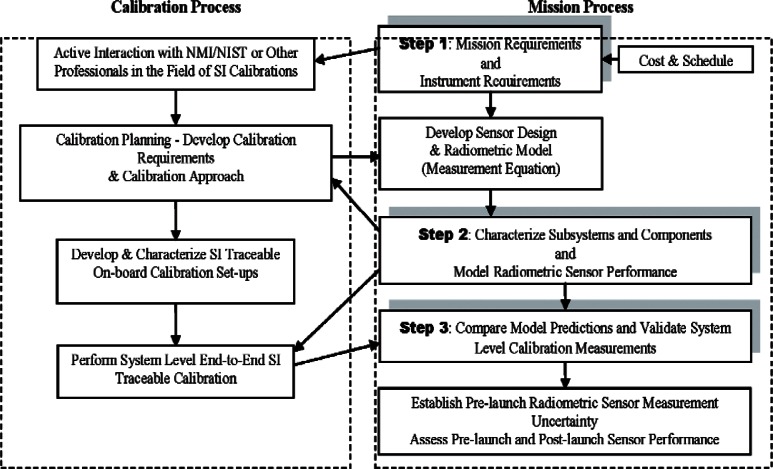
Summary steps of best practice in pre-launch calibration.

The best practice to achieve the stability and accuracy requirements for satellite optical sensors is presented as a 3 step process. Figure 1 shows the three step process.

**Step 1**: The first step is to determine the mission and calibration requirements. The mission requirements are determined by the project scientists for the type of measurements to be made. It is ideal to have radiometric experts from National Metrology Institutes (NMIs) such as NIST or other professionals active in the field for SI calibrations involved in the deliberations on radiometric accuracy requirements and availability of SI standards for calibrations.

For example, the variables in Tables 1 and 2 are linked in their role in the energetics of the climate system. If we consider one specific requirement, accurate measurements of solar irradiance are important to defining climate radiative forcing, and its accuracy requirements are specified in those tables in that context. Deliberations at the workshop in November 2002 [1] between climate modelers, calibration experts, and principal investigators of various satellite missions resulted in development of those requirements. Stringent requirements for climate demand improvement of capabilities at the NMIs to provide SI traceable standards, component characterization and system level performance validation tasks to meet those requirements for pre-launch calibrations. The mission requirements are generally specified at the science product level, while the flowed down instrument measurement requirements can be specified by either sensor scientists or instrument contractors reviewed by sensor scientists. Again, the involvement of experts from NMIs in the calibration planning with mission scientists will help to specify calibration requirements and approaches for testing SI traceability in the requisition for proposals. Interaction between NIST radiometric experts and NASA project scientists took place (although not as ideally as suggested here) for the Earth Observing System (EOS) instruments in various Platforms and provided rich experience with lessons learned for dealing with future missions. Currently similar interaction is being actively pursued with the Visible/Infrared Imager Radiometer Suite (VIIRS) and Cross-track Infrared Sounder (CrIS) instruments for the National Polar-orbiting Operational Environmental Satellite System (NPOESS) Preparatory Project (NPP). Also, interaction with NIST for the Advanced Baseline Imager (ABI) instrument in the Geostationary Operational Environmental Satellite Series (GOES-R) program of NOAA and NASA has been established. An active interaction has been initiated with NIST for the incubator projects for the future Climate Absolute Radiance and Refractivity Observations (CLARREO) mission at NASA.

The selection of SI traceable transfer radiometers from NIST depends upon the instrument accuracy requirements. Careful attention is to be paid seeking advice from experts in Radiometry at NMIs or elsewhere to develop sensor specifications based on the instrument accuracy requirements to meet mission science goals. Often this had not been the case and the sensor specifications were vague. For example, let us examine a sensor specification like “absolute radiance accuracy < 5 % required.” Such a statement without giving a coverage factor will be very vague and prone to different interpretations. In other words, is this < 5 % at coverage factor, *k* = 1, *k* = 2 or *k* = 3 level? The choice of the SI traceable transfer standard will be different for different interpretations of the uncertainty requirement for the sensor. For example, if the required level of confidence is 68.3 % corresponding to the interpretation of *k* = 1 coverage factor for the accuracy requirement of 5 % for pre-launch calibration, the requirement on the choice of the transfer standard is not very stringent. There is more flexibility in the distribution of the uncertainty in the error budget for the choice of the transfer standard and satisfy the accuracy requirement. Generally transfer standards having standard uncertainties (*k* = 1) above 2 % to 3 % are available to the industry. If the interpretation of the 5 % accuracy requirement is for coverage factor of *k* = 2 or above, standards with uncertainties below 1 % level (*k* = 1) will be required for calibration. Acquiring standards with uncertainties of 1 % or below (*k* = 1) is challenging and they may be only available at NMIs. The Step 1 shown in Fig. 1 recommends to draw expertise from NMIs and professionals in the field to develop the sensor specifications and calibration strategies that satisfy the mission requirements set by the project scientists.

**Step 2:** The component and subsystem characterization and modeling the sensor performance is the next step. As discussed in Ref. 5, characterization involves determining the component and sub-system level instrumentation responses for various operating and viewing conditions on orbit emulated in the laboratory. The sensor performance is modeled based on the sensor measurement equation. It describes all the influencing parameters on the sensor responsivity. The influencing parameters are of broadly radiometric, spectral and spatial categories. The radiometric detector characteristics, like linearity, stability, and cross talk; spectral characteristics such as responsivity, stability and accuracy; and spatial characteristics such as pointing, spatial and angular responsivity etc., are to be characterized. The spectral transmission of filters if used is very important to be characterized at operating temperatures. The mirror reflectivity and its angular dependence is also very important to be characterized. It is best to follow the axiom “Test as you fly.” That means it is important to have these characterizations performed at the environmental conditions such as temperature and vacuum as will be on orbit. However, cost and schedule are to be evaluated and characterizations are to be planned accordingly to meet the requirements. Often NMIs like NIST are well equipped to perform critical component evaluations and subsystem testing independently to confirm the sensor model, corrections and uncertainties. It is highly recommended to take advantage of such capabilities and expertise to get critical measurements done and gain high degree of confidence in building the sensor model. There are standard measurement equations that are given in Ref. 5 for the measurement of radiance, irradiance, or BRDF. As an example the measurement equation of a sensor measuring radiance in digital units can be written in a simplified equation

(1) $$DN_{i,j}G\cdot A_{i,j}\cdot \Omega \cdot L(\lambda )\cdot \Delta \lambda \cdot \eta \cdot t\cdot \tau$$

where *DN_i_,j* is the digital number output by instrument detector *i* in band *j*, *G* is the instrument detector plus digitization gain, *L*(λ) is the spectral radiance at the instrument entrance aperture, *A_i_,j* is the area of detector *i* in band *j*, Ω is the instrument acceptance solid angle, Δλ is the bandwidth, *η* is the detector quantum efficiency in electrons per incident photon, *t* is the integration time, τ is the instrument optical transmission. Instrument response non-linearity, background, focal plane temperature effects, and response versus scan angle effects are not shown in Eq. (1). These quantities are determined in pre-launch instrument characterization tests and are incorporated in instrument radiometric models and in the production of measured radiances.

Eq. (1) can be re-written as

(2) $$L(\lambda )=DN_{i,j}\cdot m$$

where

(3) $$m=\frac{1}{G\cdot A_{i,j}\cdot \Omega \cdot \Delta \lambda \cdot \eta \cdot t\cdot \tau }$$

is the inverse of the product of the instrument responsivity and gain.

**Step 3:** To compare model predictions and validate system level calibration measurements, *m* is determined pre-launch for an end-to-end remote sensing instrument by viewing uniform sources of known radiance, such as well-characterized and calibrated integrating sphere sources and blackbodies. The characterization of integrating spheres and blackbodies using SI traceable standards at NIST has been the hallmark of interaction between NIST and NASA for many of the EOS instruments including the Sea-viewing Wide Field-of-view Sensor (SeaWiFS) and the Moderate Resolution Imaging Spectroradiometer (MODIS) pre-launch sensor level calibrations. Such interactions also took place between NIST and NOAA in the past and lessons learned will be discussed in later sections of this report.

The quantity, *m*, can also be determined pre-launch through component and subsystem characterization measurements of quantities such as mirror reflectance, polarization responsivity, spectral radiance responsivity. These subsystem level characterization measurements are used as input to instrument radiometric sensor models used to validate the system level pre-launch calibration and in the calculation of instrument measurement uncertainty as shown as the final result of the best practice.

The quantity, *m*, in Eq. (3) is monitored on-orbit using stable, uniform on-board sources of known radiance. Again, on-board blackbody sources or artifacts like solar diffusers for BRDF measurements are to be developed and characterized as SI traceable standards using the expertise at NMIs like NIST as identified in Fig. 1 in Steps 2 and 3 of the best practice, which are further elaborated in Sec. 2.2 below.

### 2.2 Pre-Launch Preparation for Post-Launch Sensor Performance Assessments

Preparation for post-launch assessments of measurements and uncertainties is part of the best practice that is to be simultaneously undertaken during pre-launch preparations. A very important aspect of pre-launch testing is determining how to prove instrument stability under on-orbit conditions. Stability is often specified for long periods (Tables 1 and 2) such as mission lifetimes. It is not possible to test that long. So a “minimally acceptable” criterion should be developed during calibration planning and sufficient time should be allocated for this pre-launch activity to take place during the final phase of system level end to end calibration.

#### 2.2.1 Plan for Component Performance Reassessments—More on Step 2

One of the lessons learned at NIST in previous interactions with NASA and NOAA is that some of the sensor data problems on orbit could not be isolated fully because no duplicates or even samples of components were available for reexamination. Duplicates of filters, apertures, mirror samples, diffusers etc., are very valuable to have for reexamination at the metrology laboratories where high accuracy data can be obtained simulating the space environment and conditions of on-orbit operation to sort out data discrepancies. For example, the band edge wavelength of filter transmission is temperature dependent and it could be re-measured to understand on-orbit data. At NOAA, in the case of both GOES sounder on GOES – N and High Resolution Infrared Sounder (HIRS) on Polar Operational Environmental Satellites (POES) NOAA – N programs, a large discrepancy—as high as 6 K—was observed between the measured radiance of an on-orbit blackbody and that calculated using the pre-launch vendor-supplied spectral response function (SRF) of the sensor. This affected the on-orbit product retrieval and assimilation of Numerical Weather Prediction Models because the atmospheric quantity of interest is determined by varying it to make calculated radiances match with observed atmospheric radiances. The calculated radiance is essentially a convolution of the SRF with the monochromatic radiances from radiative transfer computation. Therefore, as a first step NOAA employed NIST to make independent measurements of SRFs of witness samples of filters of on-orbit GOES sounders. In the affected channels of GOES – 8 and GOES – 10 sounders, NIST measurements done at the on-orbit operational temperature conditions disagreed with SRFs in use by NOAA and also were found to be more consistent with on-orbit radiance observations at known blackbody temperatures, thus explaining the possible discrepancy [6]. However, the NIST measurements on witness filters were so different compared to those used at NOAA, the vendor expressed doubts on the witness samples as being authentic. A similar investigation was carried out on HIRS filters to compare vendor measurements and NIST measurements. Again, there were noticeable discrepancies and NOAA analysis showed such discrepancies affect product retrievals and their inferences on weather prediction models. As a lesson learned from this interaction, it is essential to have SRFs measured at simulated on-orbit operating conditions and they should be independently verified with authentic witness samples. In another program at NASA, the only best representative apertures of a sensor on orbit were lost in the shipment to NIST, compromising the results of a comparison of aperture area determinations among similar sensors on orbit. So one simple best practice based on all these lessons learned is that each satellite mission at least should require duplicates of critical components of their radiometric instruments for future on-orbit data reassessments. Furthermore, this best practice guideline could be extended to the manufacturing of the key instrument subsystems (in particular filters), to have instrument representatives in the manufacturing facility to closely monitor the component testing and acquire authentic duplicates of space hardware.

#### 2.2.2 Post-Launch and Pre-Launch Validation and SI Traceability—More on Step 3

Post-launch: Part of the overarching principles advocated by the workshop report [1] for high quality climate observations is to arrange for production and analysis of each Climate Data Record (CDR) independently by at least two sources. It goes on to say “Not only instruments, but also analysis algorithms and code require validation and independent confirmation.” This is because that confidence in the quantitative value of a geophysical parameter will be achieved only when different systems, different techniques produce the same value (within their combined measurement uncertainties). Comparisons of the results by different sources should reveal the flaws in particular sources to be corrected by their advocates for improving the confidence on their systems and techniques to produce high quality data. This process is broadly called as validation and is defined by the Working Group on Calibration and Validation (WGCV) of the Committee on Earth Observing Satellites (CEOS). The definition goes as “the process of assessing, by independent means the quality of the data products derived from the system outputs [7].” Traditionally it is carried out post launch through ground “truth” campaigns where different satellite systems compared their measurements on ground “truth” sites. This process of validation requires that the ground truth system is proven to be capable of credible and well proven data set on the ground “truth” site. So post launch validation should be planned using land sites of known radiometric characterization. For instruments like the Advanced Very High Resolution Radiometer (AVHRR) the ground “truth” sites essentially provide what is called vicarious calibration. For satellite sensor like the SeaWIFS the instrument calibration that began in the laboratory is continuing through vicarious calibration by comparison of data retrievals to in-water, ship, and airborne sensors to adjust instrument gains. The WGCV of CEOS is identifying suitable sites and their characteristics for on orbit sensor validations and vicarious calibrations for sensors across the world [7]. CEOS WGCV members are working with NMIs like NIST in U.S.A. and the National Physical Laboratory (NPL) in Britain in this effort. One of such sites selected is the moon as an on-orbit stability monitor for the Visible and near IR spectral region up to 2.5 micrometers. The SeaWIFS and MODIS sensors currently on-orbit have been successfully viewing the moon as a stability monitor. One of the recommendations of the ASIC3 workshop is that necessary lunar observations are to be carried out to make the moon an SI traceable absolute source for on-orbit satellite calibrations [2].

There are programs at NASA and NOAA to provide high altitude aerial platforms with radiometrically calibrated sensors for validation of satellite sensor data by simultaneously observing the satellite sensor footprint of earth’s atmosphere. The University of Wisconsin Scanning Hyperspectral Imaging Spectrometer (HIS) is an example [8].

**Fig. 2:**
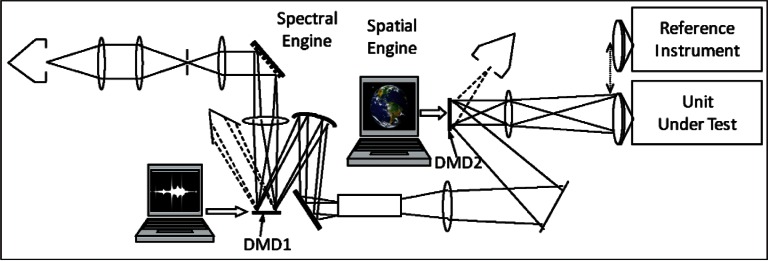
Digital Micromirror Device (DMD) projector technology to project appropriate scenes that are radiometrically calibrated using NIST standards.

Pre-launch: In following the various steps of best practice in Fig. 1, one should always be looking into new research methods and other advancements of technology to help improve the uncertainties. Although not yet proven for satellite sensor prelaunch validation activity, it is becoming possible in the laboratory to project spatially complex scenes that are radio-metrically calibrated [9]. It is achieved by using a light source and a Digital Micromirror Device (DMD)^2^ to project a scene of interest as shown in Fig. 2. The spectral engine and the spatial engine are two DMD projectors independently illuminated by light sources and controlled to project appropriate combination of the spectral features and the spatial features. There are algorithms available now in the literature to develop appropriate combination of basis spectra to project the real scene of interest.

High quality image data could be projected to the sensor and thus preflight validation could be emulated with on-orbit sensor data samples. As the accuracies of this validation equipment improve, such an exercise could help evaluate the sensor performance more realistically [10].

#### 2.2.3 On-Orbit Inter-Comparisons and SI Traceability

It is best to have inter-comparisons of similar sensors on orbit to assess consistency in data products and sensor performance. Such inter-comparisons are possible when both sensors being intercompared are SI traceable on orbit. Intercomparison of on-orbit sensors has become possible with the technique of Simultaneous Nadir Observations (SNO) when both satellites observe the same foot-print at nearly the same time they cross each other in their orbits [11]. In order to bring self-consistency and intercalibration of sensors across the world, a group called Global Space-based Inter-Calibration System (GSICS) formed and is actively pursuing SI traceability for intercalibrations working with NMIs like NIST. Intercomparisons may show good agreement or disagreement between sensors in their radiance measurements. In either case, lessons will be learned on possible systematic effects that are currently ignored or neglected. As the true value of the measurand on orbit will always be an unknown quantity, the accuracy of the measurement can best be assessed by combining the results from different sensors and calculating the uncertainty of that Combined Reference Value (CRV) based on the individual sensor data [12]. The CRV and the estimate of its uncertainty in the time series will allow scientists to look into methods to minimize uncertainties and achieve the stated accuracy requirements by using the lessons learned through intercomparisons.

## 3. Secondary Laboratories for SI Traceability and Role of NMI like NIST

For many remote sensing projects, the extensive, direct incorporation of NMI calibration experts and equipment is impractical. In these cases and depending on target calibration and characterization uncertainties, these projects are served by NMI-provided calibration standards, by the project's internal calibration and characterization programs, and by other industrial, academic, or government secondary standards facilities. For many manufacturers of satellite instruments this is the case. In these cases, the satellite manufacturer, industrial, academic, and government facilities are secondary standards laboratories themselves, establishing or providing SI traceability through NMI-provided calibration artifacts coupled with sound measurement methodologies and techniques. For these laboratories, the development and maintenance of an internal calibration program require a substantial capital investment. This is particularly true for manufacturers of smaller remote sensing instruments used in field validation work and for projects that provide remote sensing measurements in the field. In the case of satellite remote sensing programs, independent field measurements are a critical source of calibration and validation measurements, that is, of ground truth for the post launch operation of the satellites. Here the SI traceability of the secondary standards laboratories used by validation projects complements the SI traceability of the pre-launch calibration of the satellite sensors. Both are necessary for long-term, satellite-based, climate-quality remote sensing programs.

## 4. UV, VIS/NIR and IR Standards at NIST for SI Traceability

The optical radiation measurements are generally referenced to one of two SI scales: optical power in watts or thermodynamic temperature in kelvin. The SI unit, electrical watt is tied to the optical watt via electrical substitution achieved by the use of cryogenic radiometers. The temperature scale is derived from the triple point of water. The SI unit kelvin is defined as 1/273.16 of the thermodynamic temperature of the triple point of water. The temperature at that point is defined as 0.01 °C in the commonly used Celsius scale.

### 4.1 Primary Optical Watt Radiometer (POWR)

**Fig. 3:**
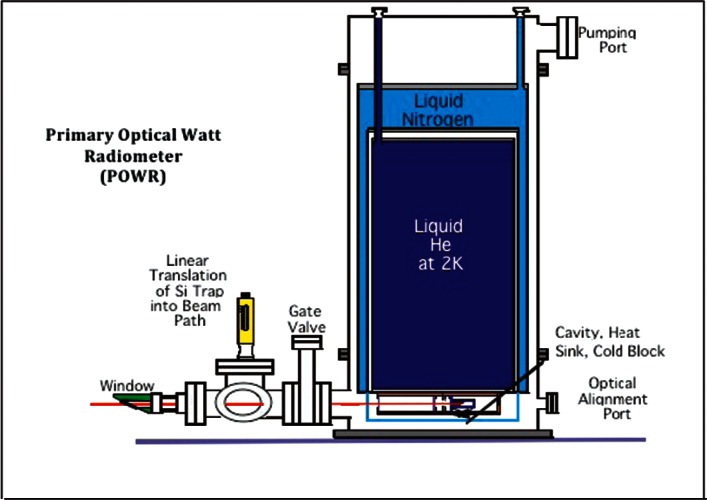
Schematic of NIST primary standard for optical power measurements, Primary Optical Watt Radiometer, POWR.

At NIST, the Optical Technology Division realizes and maintains the unit of optical power (watt) using a custom built state of the art electrical substitution radiometer, called the Primary Optical Watt Radiometer (POWR), as shown schematically in Fig. 3. It is operated at 2 K or 4.2 K to minimize background effects. Its dynamic range is 1 μW to 1 mW in measuring optical power from intensity stabilized lasers. The standard uncertainty achieved in POWR measurements is at 0.02 % level [13]. Silicon trap detectors which have absolute quantum efficiency close to unity are used to transfer the power scale from POWR to other cryogenic radiometers or detectors. The standard uncertainty for such transfers is 0.02 % to 0.04 % [13].

### 4.2 Irradiance and Radiance Scales

The irradiance, E (W / cm^2^) and the radiance L (W/cm^2^ sr) can be derived from the measurement of radiant power or from the Planck’s law for the radiance of a perfect blackbody. The former method of scale realization is called as detector based and the later is called as source based. NIST realizes these scales both ways and the following sections describe the corresponding facilities at NIST.

#### 4.2.1 Spectral Irradiance and Radiance Responsivity Calibrations Using Uniform Sources (SIRCUS)

**Fig. 4:**
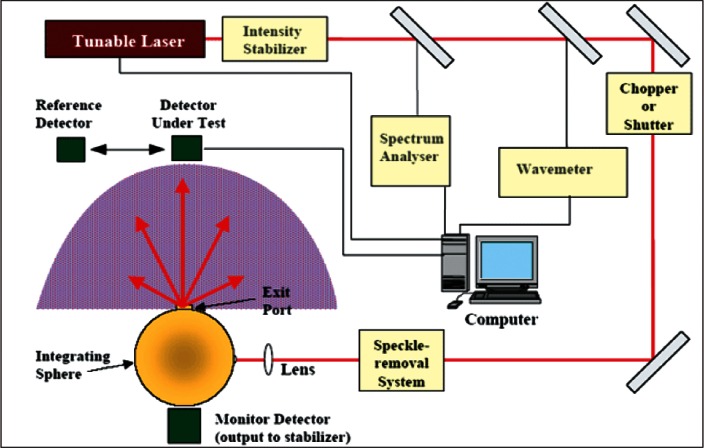
Spectral Irradiance and Radiance Responsivity Calibrations using Uniform Sources (SIRCUS) Facility at NIST.

In order to calibrate detectors and radiometers for spectral irradiance responsivity and spectral radiance responsivity from the UV to the IR, a facility called SIRCUS is available at NIST as a schematic diagram of key components of the facility is shown in Fig. 4. Light from a tunable laser illuminates the integrating sphere. The output of the integrating sphere through a precision aperture is used for irradiance and radiance calibrations. In this facility, tunable lasers covering the wavelength range from 210 nm to 5.3 μm are coupled to integrating spheres to produce either uniform irradiance at a reference plane or uniform radiance within the sphere exit port at high levels. Detectors are calibrated directly against reference standards such as the trap detectors referenced earlier. Lasers ultimately determine the spectral coverage available at SIRCUS, while the uncertainties achievable ultimately depend on the quality of the transfer standard detectors. Currently, uncertainties are in the 0.1 % level [14] for the visible and at few percent levels for the infrared detector calibrations [15]. The SIRCUS covers the UV-Vis-NIR spectral region with one set of lasers and an integrating sphere with Spectralon coating, and the other set of tunable lasers for the IR from 700 nm to 5.3 μm using a diffuse gold coated integrating sphere. Also discrete lasers extend the spectral coverage to 10 μm. A portable, table-top, tunable laser system, complete with integrating spheres and transfer standard detectors, called Traveling SIRCUS, to cover UV-Vis-NIR region is available at NIST to visit customer facilities and provide on-site calibrations. It has been to many customer facilities, including NASA, NOAA, and United States Geological Survey (USGS) sites to characterize instruments.

#### 4.2.2 Facility for Automated Spectral Irradiance and Radiance Calibrations (FASCAL)

The FASCAL facility derives and maintains its radiance scale from the absolute radiance of a gold fixed-point blackbody whose temperature is held at the freezing point of gold (1064.18 °C) and whose radiance follows Planck Radiation Law. The gold fixed-point black-body at NIST is used to realize and disseminate the 1990 NIST Radiance Temperature Scale (1990 NIST). Experiments at NIST established that the 1990 NIST Spectral Radiance Scale derived from the gold fixed-point blackbody compared well with the radiance scale derived from optical watt [16]. The FASCAL facility uses a CARY 14 spectroradiometer for radiance calibrations with respect to a working standard variable-temperature blackbody (VTBB) whose radiance temperature is derived from the gold fixed-point blackbody. The spectroradiometer covers the wavelength range from 225 nm to 2500 nm with suitable detectors. A S-20 response photomultiplier tube is used to cover 200 nm to 900 nm and a 4 stage TE cooled InGaAs photodiode is used to cover 800 nm to 2500 nm.

For remote sensing applications in the reflected solar spectral region, an integrating sphere, the NIST Portable Radiance (NPR) source, was developed by NIST as part of the EOS collaboration [17]. The NPR is 30 cm in diameter with a 10 cm diameter exit aperture. It is used during EOS comparison activities to verify the stability of the NIST absolute radiometers and to provide a calibration of the NIST transfer radiometers. There are four baffled lamps arranged symmetrically about the inner lip of the exit aperture, and different radiance levels are achieved by operating with one to four lamps illuminated. The NPR is calibrated on FASCAL; the relative expanded uncertainties (*k* = 2) in spectral radiance for the NPR are 0.5 % in the visible, increasing to 1.0 % at 300 nm and 2.5 % at 2400 nm (all *k* = 1). Because it is a stable source with two monitor photodiodes and the radiance assignment is made directly using the national radiance standards maintained at NIST, the NPR can also be used to assess the accuracy of the radiance calibration of participants’ radiometers. Such an assessment is important because the common method of establishing spectral radiance traceability to NIST involves the spectral irradiance standards (i.e., the FEL lamps) and reflectance standards. Thus the NPR provides a direct comparison to NIST’s spectral radiance scale.

#### 4.2.3 Advanced Infrared Radiometry and Imaging (AIRI) Facility

The kelvin thermodynamic temperature scale is realized through melting points of various pure metals as defined in the International Temperature Scale of 1990 (ITS-90) [19]. At NIST these blackbodies are used to calibrate standard platinum resistance thermometers (SPRTs) to cover the temperature range from 15 °C to 170 °C and standard gold-platinum thermocouples to cover the range from 400 °C to 900 °C. These temperature sensors are used in the ideal blackbodies such as the water bath, cesium heat pipe, and others to provide SI traceable calibrations of radiance temperature and radiance using a transfer standard spectroradiometer. These heat pipe blackbodies and various fixed point blackbodies for radiance temperature measurements and radiometers and pyrometers at the Advanced Infrared Radiometry and Imaging (AIRI) Facility at NIST are shown in Figs. 6a. and 6b. The AIRI facility allows realization of uncertainties in the 50 mK range in the radiance temperature calibrations.

#### 4.2.4 Synchrotron/D2 Lamps

Synchrotron radiation has been recognized as an absolute radiation as its characteristics are calculable by the Schwinger equation just as the blackbody radiation is calculable by using Planck equation. In the UV and shorter wavelength regions, synchrotron radiation from a storage ring has the highest accuracy in calculable irradiance among all currently available source standards. NIST has a synchrotron storage ring, the Synchrotron Ultraviolet Radiation Facility III (SURF III), with two beam lines, Beam line 3 and Beam line 4, dedicated to calibrations in the UV. Beam line 4 provides calibrations of UV detectors. Beam line 3 provides source based radiometry and is called the Facility for Irradiance Calibration Using Synchrotron, FICUS. It provides calibrations from 200 nm to 400 nm [20]. The combined relative uncertainty for FICUS is 0.7 % (*k* = 2). Standard light sources such as D2 lamps used in remote sensing laboratories and elsewhere are calibrated for irradiance at FICUS. The relative combined uncertainty for D2 lamp calibrations is 1.2 % (*k* = 2).

## 5. Transfer Standard Radiometers at NIST

Radiometers have been built at NIST and calibration protocols have been established for deployment at customer sites to provide SI traceable calibrations or validations. Here we describe transfer radiometers for the reflected solar, and the thermal IR spectral regions.

### 5.1 Transfer Radiometers for the Reflected Solar Spectral Region

#### 5.1.1 Ultraviolet (UV)

The UV spectral region is significant to the ozone and downwelling solar surface irradiance communities. Two types of instruments have been used over the years—a portable scanning monochromator and a UV filter radiometer. The NIST scanning monochromator is a 0.125 m f/4.7 Fastie-Ebert double monochromator with 0.02 nm wavelength accuracy [21]. The configuration is versatile, allowing the instrument to be used in comparisons of spectral irradiance scales within NIST or between NIST and other NMIs [22], participation in field comparisons of UV downwelling surface spectral irradiance [23], in EOS comparisons of spectral radiance standards [24], and for calibrating sources at vendor facilities [25]. The NIST UV spectroradiometer (UVSR) is operated as a transfer radiometer in the strictest sense, that is, the reference source and the unknown source are compared sequentially during the measurement activity. For field work, this means a stable, calibrated reference source such as the NPR must be available. The core elements of the UV filter radiometer are a silicon trap detector, a five position temperature stabilized filter wheel, and a pair of precision apertures separated by a fixed distance to define the throughput [26]. The narrow bandwidth filters were selected to coincide with ozone retrieval algorithms. The UVFR has been used to calibrate and monitor sources at vendor facilities [27] and to validate spectral radiance scales in the UV [25, 27]. To date, the spectral transmittance data is based on values supplied by the manufacturer, which increases the uncertainty in the validation exercises.

#### 5.1.2 Visible and Near Infrared (Vis/NIR)

The Vis/NIR spectral region is the basis of many retrieval algorithms that are used to derive ocean, land, and atmosphere products such as chlorophyll concentration, leaf area index, aerosol optical thickness, and so on. In support of the SeaWiFS Project, NIST designed and delivered a six channel filter radiometer, the SeaWiFS Transfer Radiometer (SXR) [28]. In subsequent years, the mechanical and optical configuration was improved and produced commercially by Reyer Corporation, Frederick, Maryland. Three copies were made: the Visible Transfer Radiometer (VXR) [29], the Landsat Transfer Radiometer (LXR) [30], and the SXR II [31]. The VXR is currently maintained and operated by NIST for NASA/GSFC, with original funding provided by the EOS Project Science Office. The LXR was funded by Landsat 7 and is now part of the cal/va1 activities for the Operational Land Imager (OLI) Project; the SXR and SXR II were funded by the SeaWiFS Project Office.

These four filter radiometers are essentially the same—each has six narrowband channels between 400 nm and 900 nm defined using interference filters and six silicon photodiode detectors. The instruments measure radiance. A camera lens is the foreoptic, producing an image at the field stop. Behind the field stop, six different wedge-shaped mirrors with spherical curvature focus the field stop at six image locations, where the six filter/detector elements are located. The temperature of the field stop, filters, and detectors is maintained at a constant value using thermoelectric elements. An on-axis optical system is used to align and focus these radiometers. The field-of-view is about 2.5°, and the minimum object distance is 85 cm. Two methods have been used to calibrate these filter radiometers. The first is traceable to the spectral radiance values of a reference source such as the NPR, with the necessary relative spectral responsivity functions for the channels derived from independent, system-level measurements. The second is traceable to POWR using SIRCUS. Here, the absolute spectral radiance responsivity is determined with subnanometer spacing for the in-band region of the filter transmittance response and coarser spacing over the out-of-band regions by comparison to a reference detector. The relative combined standard uncertainty of the VXR’s calibration coefficients is about 0.74 % for the NPR calibration, and 0.25 % for the SIRCUS calibration [29].

#### 5.1.3 Short-Wave Infrared (SWIR)

The spectral region from around 800 nm to 2500 nm has important applications such as surface soil and mineral mapping, cirrus cloud detection, and land and cloud properties. In collaboration with the EOS Project Office, NIST developed the EOS Shortwave infrared radiometer (SWIXR) [32] for use as a transfer radiometer in this spectral region. The EOS SWIXR is a scanning, double-grating monochromator-based instrument equipped with all-reflective input optics and a liquid-nitrogen-cooled indium antimonide (InSb) detector. The full-angle field-of-view is 5.2° and a cold filter in front of the InSb detector reduces the amount of stray light and thermal infrared background radiation incident on the detector. Order sorting filters further reduce the stray light in the system, resulting in a stray light rejection of 10^−7^. The wavelength reproducibility is 0.1 nm, and the corrected wavelength uncertainty is 0.2 nm. A chopper, three-stage trans-impedance amplifier, and a lock-in amplifier comprise the InSb detection system. The SWIXR is used as a transfer radiometer, with calibration in the field using the NPR. The relative combined standard uncertainty in the SWIXR responsivity is approximately 2 % from 1000 nm to 2200 nm, and increases slightly to 2.5 % at 2400 nm.

A summary of intercomparisons in the Vis/NIR/SWIR is given in Refs. 33 and 34. The participants included NIST, NASA/GSFC, the University of Arizona (UA), and Japan’s National Research Laboratory of Metrology (NRLM). Between February 1995 and April 2001, 10 intercomparions were held utilizing 11 different transfer radiometers [33]. The reported level of agreement was ± 1.80 % at 411 nm, ± 1.31 % at 552.5 nm, ± 1.32 % at 868.0 nm, ± 2.54 % at 1622 nm, and ± 2.81 % at 2200 nm.

### 5.2 Transfer Radiometers for the IR

#### 5.2.1 TXR

NIST developed the TXR in support of NASA’s Earth Observing System (EOS) program [35] and its deployment at the customer site is in line with the best practice advocated earlier in Sec. 2.1. The TXR measures radiance scales in the thermal-infrared spectral region for satellite sensors calibrated by extended area blackbody sources at customer sites [36]. The TXR is a portable two channel radiometer, with one channel at 5 μm using a photovoltaic InSb detector and the other at 10 μm using a photovoltaic MCT detector. It has a self-contained vacuum jacket and liquid nitrogen (LN_2_) reservoir. It can be used for radiance scale verifications of blackbody sources either in cryogenic vacuum chambers or in ambient conditions. The standard uncertainty for radiance measurements using the TXR is of the order of 0.2 % (*k* = 1) or better. In terms of radiance temperature deduced from TXR measurements for blackbodies operated between 200 K to 350 K, the uncertainty is in the 50 mK (*k* = 1) range. The TXR can be used to measure emitted radiance as well as, with special setups, the reflected radiances from the black-body. From such measurements the blackbody emissivity can be deduced. In a deployment at Raytheon Santa Barbara Remote Sensing, the TXR characterized the blackbody calibration source (BCS) that was used to calibrate NASA’s MODIS sensor. The measurements verified the emissivity to the 0.001 % level and the emitted radiance scale was found to be in agreement with the NIST scale with no corrections needed. The TXR calibration at NIST is carried out by using several methods. One method uses at the system level the ambient background water bath blackbody or a Large Area Black Body (LABB) for the absolute calibration. Another method still under development is a system-level approach using a laser-illuminated integrating sphere at the NIST SIRCUS described in Sec. 3. The TXR has been successfully deployed about six times during the past several years to several different aerospace calibration facilities for in-situ measurements of various sources in space-simulating chambers. These measurements were used to verify the infrared radiance scales currently used by several NASA, NOAA, DOE, and DOD satellite programs. The results of a deployment of TXR to the GOES calibration chamber at the contractor (ITT) site in Ft. Wayne, IN in 2001 is reviewed in Sec. 6 as an illustration of best practice.

#### 5.2.2 FTXR and MDXR

The Fourier-transform Thermal-infrared Transfer Radiometer (FTXR) is a spectroradiometer system designed to measure spectral radiance in the infrared. The original motivation for its use at NIST was to improve upon the spectral coverage of the TXR for comparisons of extended-area blackbodies such as those used to calibrate Earth-observing satellite and validation instruments. The spectral coverage of the FTXR for use in viewing such blackbodies is 800 cm^−1^ to 12000 cm^−1^ using both an MCT and an InSb detector. Both of these detectors are used at the same time, since the FTXR has two detector ports that share a common input port, and their spectra are concatenated to provide the full spectral range. It is based on a four port Michelson interferometer. It has corner cubes and flexture mounts, for a spectral resolution of roughly one cm^−1^. The scale of the FTXR can in principle be derived from the NIST Water Bath Blackbody (WBBB) rather than the on-board blackbodies. The FTXR is designed to operate in ambient environments, so it needs to look through a window to view blackbodies in vacuum chambers. In such an arrangement one has to limit observations to atmospheric transmission regions of the IR spectrum unless a very good purge arrangement is available. Also the window transmission, reflection, and stray light introduce extra systematic uncertainties. Therefore, a new radiometer called the Missile Defense Transfer Radiometer (MDXR) is under construction to mitigate these problems. The MDXR will have the capabilities of the TXR and also is equipped with a cryogenic Fourier transform spectrometer to cover the wavelength of interest, a cryogenic radiometer traceable to POWR, and a black-body calibrated at the NIST Low Background Infrared (LBIR) facility. A vacuum compatible fluid bath black-body is also under construction to provide kelvin scale calibration. The MDXR will also be self-contained to serve user facilities in both radiance and irradiance modes at the power levels that are of interest.

## 6. Examples of Best Practice

Four examples of best practice to build SI traceability for instrumentation in remote sensing programs where NIST was involved are described below. The first example was the intercomparison of the SI traceable VIS/NIR transfer radiometers at the EOS facility at Goddard Space Flight Center (GSFC) at NASA to assess the current state of the art radiance and irradiance uncertainty across the available instrumentation. The second was the historical and current account of achieving the SI traceability for the VIS/NIR bands of the Terra and Aqua satellite MODIS sensors from pre-flight to current operational scenarios. The third was the laboratory intercomparison of infrared radiometers funded by NOAA / NESDIS, EMETSAT, ESA and NASA in 2001 at the University of Miami’s Rosenstiel School of Marine and Atmospheric Science (RSMAS). The fourth one is the TXR deployment for the calibration of the blackbody in the GOES imager test chamber at ITT, Ft. Wayne, Indiana in 2001.

### 6.1 NASA/GSFC Intercomparison of VIS/NIR Transfer Radiometers

For more than two decades, NASA GSFC’s Calibration Facility (CF) has provided calibration and characterization services to a large number of ground-based, airborne, and space-borne Earth viewing sensors. Radiance and irradiance calibrations in the CF are performed in the facility’s Code 614.4 Radiometric Calibration Laboratory (RCL) and Code 613.3 Radiometric Calibration and Development Laboratory (RCDL). The primary artifacts used in these laboratories for the radiance calibration of these sensors in the UV to the SWIR, e.g., 230 nm to 2500 nm, are lamp illuminated integrating sphere sources. In April 2001, a measurement comparison was held in the RCL to validate the radiance scales assigned to the RCL 180 cm and RCDL 50.8 cm spheres, to critically examine sphere operation, sphere radiance repeatability and stability within the RCL and RCDL, and to validate the radiance measurements of a number of radiometers used in the vicarious calibration of fundamental NASA Earth viewing satellite instrument measurements. In addition, a Spectralon reflectance panel illuminated by lamps calibrated against the NIST 1990/1992 and NIST 2000 irradiance scales was measured by several radiometers to examine the effect of scale differences on their radiance measurements. This comparison was one of a series of validation activities overseen by the EOS Calibration Program to ensure consistency in the radiometric calibration accuracy of sensors used in long-term, global, climate remote-sensing.

In this comparison, the radiance scale on the CF spheres was realized using two GSFC transfer radiometers: the Shuttle Solar Backscatter Ultraviolet (SSBUV) and the 746/Integrating Sphere Irradiance Collector (ISIC) instruments. Both are scanning monochromators with radiance responsivities that are traceable to the NIST standard irradiance lamps. A number of transfer radiometers were used in an independent determination of these radiance scales: the UVSR, UVFR, VXR, SWIXR from NIST; the UA Visible/Near InfraRed (VNIR) and UA ShortWave InfraRed (SWIR) from the University of Arizona; the LXR and the SXR II from GSFC; three ASD’s, one from South Dakota State University (SDSU), one from NASA Ames, and one from GSFC; and the Research Support Instrument (RSI) Calibration Transfer Standard Radiometer System (CTSS) Multifilter Spectroradiometer (MFS). During the comparison, the radiometers made measurements over the spectral range from 300 nm to 2400 nm, spanning the UV to SWIR wavelength regions.

Four radiance sources were measured: the NPR, the GSFC Code 614.4 180 cm diameter integrating sphere, the GSFC Code 613.3 50.8 cm diameter integrating sphere, and a Spectralon panel illuminated by two NIST irradiance standard lamps, type FEL, designated F-496 and F-512, respectively.

The primary goal of the comparison was to assess the uncertainties of the spectral radiance assignments of the two GSFC spheres. A second goal was to characterize the source stability and repeatability using a newly implemented monitoring detector system on one of the GSFC spheres. The results were analyzed by using the radiance scale realized by the GSFC 746/ISIC and SSBUV spectroradiometers and the individual spectral responsivity functions for the transfer radiometers to calculate the band-averaged spectral radiance; these results were then compared to the spectral radiances measured by the instruments. The preliminary results showed that the 180 cm sphere was not stable in time for wavelengths below 550 nm but the 50.8 cm sphere was stable in this spectral region [37]. The higher than expected results for the 746/ISIC below 450 nm were later identified to be the result of uncorrected stray light in this single grating monochromator [24], and it was noted that this effect is likely affecting the results with the ASDs. Another bias that was observed was suggested to be understood in terms of the irradiance scale assigned to the FEL lamps—the NIST 2000 was not implemented on all the FEL lamps consistently, so the known differences between that scale assignment and the previous, NIST 1990/1992 assignment, would be evident. The overall results were consistent within the *k* = 2 uncertainties. One exception was the NIST SWIXR and the UA SWIR compared to the GSFC 746/ISIC on the l80 cm and 50.8 cm spheres—beyond 2000 nm the results were outside these expanded uncertainty values for the small, but not the large sphere. Also identified were likely calibration errors in some of the CTSS channels and the SDSU ASD. The results of the lamp/plaque radiance comparison were reproducible and also indicated the presence of the bias caused by using two different NIST irradiance scales [38]. This reinforced the need for irradiance standard lamps to be calibrated against the NIST 2000 detector-based irradiance scale, resulting in improved accuracy and reduced uncertainties, especially in the SWIR.

### 6.2 Terra and Acqua MODIS VIS/NIR SI Traceability

MODIS is a key instrument for the NASA’s EOS missions, currently operated onboard the Terra and Aqua spacecrafts, launched in December 1999 and May 2002, respectively. MODIS is a cross-track scanning radiometer. It collects data in 36 spectral bands, from VIS to LWIR, and at three nadir spatial resolutions: 0.25 km, 0.5 km, and 1 km. Improved over its heritage sensors, such as AVHRR and Landsat, MODIS was designed with stringent on-orbit calibration requirements: 2 % for VIS/NIR reflectance and 5 % for the radiances (all *k* = 1). Because of this, it was built with a set of state of the art on-board calibrators, including a solar diffuser (SD), a solar diffuser stability monitor (SDSM), a black-body (BB), a spectroradiometric calibration assembly (SRCA), and a space view (SV) port. The SD and DSM are used together for the VIS/NIR and SWIR calibration and BB for the thermal infrared (TIR) calibration. The SRCA is primarily used for the sensor spectral (VIS/NIR) and spatial characterization [39].

Both Terra and Aqua MODIS went through extensive preflight calibration and characterization activities. The VIS/NIR preflight radiometric calibration was made in the thermal vacuum (TV) using a spectral integrating sphere (SIS) operated at multiple radiance levels (or SIS lamp combinations) external to the sphere through a chamber window. The MODIS instrument vendor’s calibration of the SIS traceable to NIST was validated using the NIST VXR and SWIXR at the instrument vendor’s facility. In addition, radiance measurements of the SIS at the instrument vendor’s facility were made by the GSFC 746/SIC, the UA VNIR and SWIR radiometers, the GSFC LXR, and radiometers from Japan’s National Research Laboratory for Metrology (NRLM, now Advanced Industrial Science and Technology (AIST)). To simulate sensor on-orbit operational conditions, the thermal vacuum was operated at three different instrument temperatures: cold, nominal, and hot, and the sensor characterized with its primary and redundant electronics. Key parameters examined during MODIS preflight TV calibration and characterization included VIS/NIR detector response (gain), signal-to-noise ratio (SNR), nonlinearity, dynamic range, and detector gain’s sensitivity to the instrument temperatures. Other system level calibrations for the VIS/NIR spectral bands were made at ambient environment, including sensor relative spectral response (RSR), polarization sensitivity, band-to-band registration, and response versus scan angle [40].

**Fig. 7:**
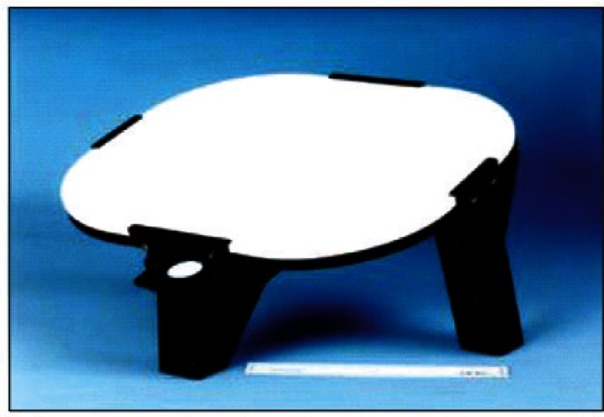
MODIS solar diffuser (SD) panel.

**Fig. 8:**
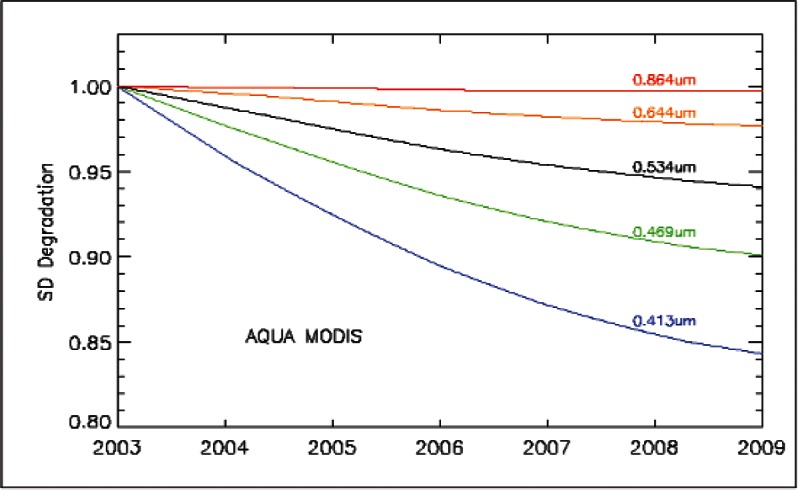
Aqua MODIS solar diffuser degradation (2003 to 2008).

MODIS VIS/NIR on-orbit calibration is reflectance based with reference to the bi-directional reflectance factor (BRF) of its on-board solar diffuser (SD), as shown in Fig. 7. The SD BRF characterization was made pre-launch by the instrument vendor using reference samples traceable directly to NIST. Its on-orbit degradation is tracked by the on-board SDSM at 9 wavelengths, closely matched to some of the MODIS VIS/NIR spectral bands. The SDSM functions as a radiometer that ratios alternate signals, making alternate observations of direct sunlight and the sunlight diffusely reflected from the SD. The SDSM is operated during each scheduled SD calibration event. Figure 8 illustrates the on-orbit degradation of the SD on Aqua MODIS at 0.41 μm, 0.46 μm, and 0.53 μm. The SD degradation is much smaller at other longer wavelengths, 0.64 μm and 0.86 μm. In addition to SD/SDSM calibrations, both Terra and Aqua MODIS use monthly lunar observations to monitor the VIS/NIR radiometric calibration stability [41].

### 6.3 Miami Workshop 2001

The intercomparison workshop that NIST participated in at the University of Miami’s Rosenstiel School of Marine and Atmospheric Science (RSMAS) in 2001 dealt with the intercomparison of blackbodies used to calibrate radiometers that are deployed on ships to measure sea surface temperature [42]. As NIST employed the TXR for this purpose, this process provided an independent experimental check of the SI traceability of sea surface temperature measurements. Such intercomparisons are highly recommended in this paper as a best practice for SI traceability.

The NIST TXR described earlier in Sec. 4.2.1 was employed [36] in reasonably controlled laboratory conditions to view several cavity blackbodies and measure the brightness temperature of each. The laboratory blackbodies were five in total, one a reference blackbody, the NIST water bath blackbody (WBBB), and four other participating blackbodies (BB): The RSMAS BB, the Jet Propulsion Laboratory (JPL) BB, the Combined Action for the Study of the Ocean Thermal Skin (CASOTS) Rutherford Appleton Laboratory (RAL) BB, and the CASOTS Southampton Oceanography Centre (SOC) BB. All of these were operated independently of each other in the same laboratory at RSMAS during the workshop. Each BB consisted of a conical metal cavity with a black coating on the inside and each was surrounded on the outside by its own stirred fluid bath to improve temperature uniformity. They each had a calibrated thermometer located in the stirred bath, which was used to determine the temperature of the cavity. All cavity exit apertures were of the order of 10 cm to 11 cm in diameter, and all BBs were designed to be horizontally emitting. Beyond these general similarities, these five BBs can be classified into two groups depending on whether the bath temperature has active control or not. The NIST water bath blackbody (WBBB) and the RSMAS BB have active temperature control of the bath and essentially follow a design described previously [43]. The JPL BB, the CASOTS RAL BB, and the CASOTS SOC BB do not have active temperature control and follow another general design described previously [44]. The black-bodies intercompared were designed so that the emissivity is as high as possible even with a relatively large aperture diameter of about 10 cm to 11 cm. The TXR target spot diameter was about 3 cm, so it under-viewed these apertures. The TXR was placed sufficiently close to each BB under test such that the TXR 30 mrad field of view was overfilled by the BB aperture.

**Fig. 9:**
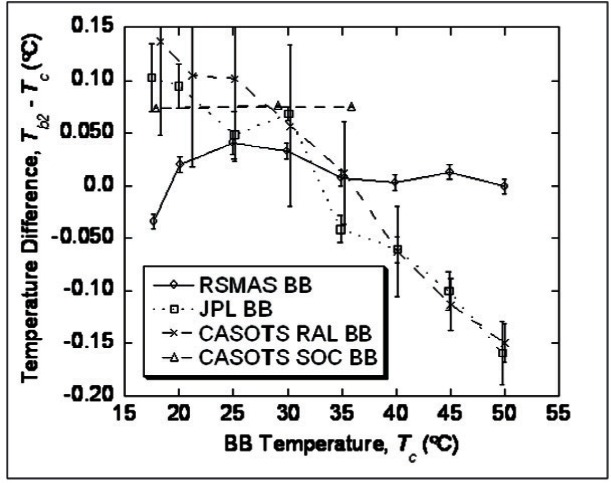
Comparisons are brightness temperature. The symbols are from the mean values of data points averaged over the last 100 seconds of each plateau of the temperature setting for each blackbody. The error bars are the standard deviation of the values over this time interval.

The results of the intercomparison are shown in Fig. 9 in terms of 10 μm TXR brightness temperature (*T_b_*_2_) derived from its calibration with the NIST waterbath blackbody minus contact temperature (*T*_c_) for the test blackbody as function of test blackbody temperature for all four participating test blackbodies: RSMAS BB, JPL BB, CASOTS RAL BB, and CASOTS SOC BB. The *T*_c_ values for each BB are from the user’s choice of thermometer placed in the BB water bath. Averages over the last 200 seconds of each plateau interval are reported for all but the CASOTS SOC comparison, and the error bars represent the standard deviation of the 100 readings of this last, most stable interval on the plateau. The RSMAS BB error bars are much lower, down at the stability level of the TXR, since the RSMAS BB was under active temperature control and so its plateaus were very flat with time. For the other blackbodies, the lack of active temperature control over the plateau caused temperature drift to dominate the uncertainty, hence the larger error bars. Plotting only instantaneous points, rather than interval averages, gives similar results. Figure 9 also lets us infer that the RSMAS BB 10 μm brightness temperature agrees with that of the NIST water bath black-body, which is the reference blackbody for TXR calibration over the entire range of temperatures studied, to within the ± 0.05 °C (*k* = 2) uncertainty of the TXR.

The CASOTS RAL and JPL blackbodies did not agree at temperatures away from ambient, although they did agree to within ± 0.1 °C as long as they were near ambient. Effective emissivity values relative to the NIST water bath blackbody were near 0.991 at 10 μm for both of these blackbodies. The CASOTS SOC blackbody was not measured carefully enough to draw any definite conclusions. Careful use of these black-body targets to calibrate ship-based radiometers used in the validation of satellite-derived skin sea-surface temperatures could therefore result in validation data sets that have uncertainties within ± 0.1 °C. This inter-comparison also demonstrated some of the verification capabilities that are now available to the environmental remote sensing community with the use of the NIST TXR.

### 6.4 TXR Deployment to ITT for GOES Imager

The NIST thermal-infrared transfer radiometer (TXR) was deployed in the GOES Imager calibration chamber in July of 2001 and performed radiometric measurements of the calibration targets used to calibrate GOES Imager instruments. The GOES Imager emissive band pre-flight absolute calibration is generically similar to most radiometric calibration exercises in that it is based on measurements of the instrument response to two blackbodies held at different temperatures in a space-simulating cryogenic vacuum chamber. Traditionally, the radiance entering the sensor aperture is modeled, starting with the temperature sensors in the warm blackbody, here called the Earth Calibration Target (ECT). As the measurements are performed in a significant thermal-infrared background, correction for background-induced offsets is made by subtracting the response to a liquid-nitrogen temperature blackbody, here called the Space Clamp Target (SCT).

It is believed that the honeycomb surface of the ECT can support substantial temperature gradients forced by the thermal-infrared background. The GOES calibration model makes a correction for the non-ideal behavior of the ECT. The TXR was deployed to provide independent quantitative verification of this model through careful radiance measurements from the ECT and the SCT.

Within the GOES Imager chamber, the TXR was mounted on a platform in a specially-constructed GOES instrument simulator. The instrument simulator was an aluminum frame structure which supported multilayer insulation. The simulator was designed by ITT to look from the outside as much like a GOES Imager instrument as possible, except that it had the TXR inside instead of an actual GOES instrument. The instrument simulator included an optical port baffle, just as the GOES imager does when in the chamber. This is normally run at one of three temperatures during GOES instrument testing: Mission Low (ML), Mission Nominal (MN), and Mission High (MH). For the TXR deployment, the optical port baffle was run at only the two lowest temperatures: ML and MN. The radiative cooler patch panels, used with a real GOES instrument to provide cooling for the GOES radiative coolers, were also adjusted between ML and MN conditions to the same temperatures that they are set at for real GOES instrument testing. This was all done so as to simulate the radiometric environment existing during a typical GOES instrument calibration.

**Fig. 10:**
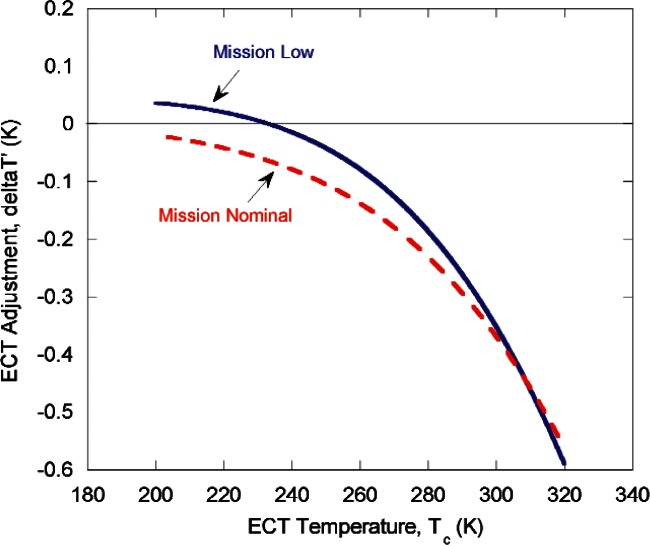
The recommended ECT adjustment curves, delta *T*' vs. *T*_c_, with *T*_c_ defined as the average of all 13 ECT sensors. Correction of the ECT to blackbody radiance requires only these curves and no emissivity adjustment. This is not to say that the emissivity of the ECT was unity. Rather, a value of unity has been historically used for the ECT in the GOES program, so these curves lump the combined effects of non-unity emissivity and temperature error into one temperature dependent parameter in order to make the required corrections simpler to implement using the existing GOES calibration algorithms.

There were two main types of tests: TXR response measurements of its internal calibration source (CS), and TXR response measurements to the ECT (or SCT). The response data were collected and analyzed as band-integrated radiances and compared to that expected for an ideal blackbody. An analysis procedure was developed that enabled parameterization of the results in terms of a non-unity emissivity and a temperature gradient in the GOES Earth Calibration Target (ECT). The model was used to compute the correction to be made to the ECT temperature sensor readings so that the measured results are in agreement with the NIST radiometric scale. Values for the true temperature and ECT emissivity at standard uncertainty levels below 0.1 K and below 0.2 %, respectively, were obtained. Recommended values for ECT temperature correction as shown in Fig. 10, were computed based upon a fit of the model to the data. The data agreed qualitatively with the expectations. These data, in the form of an electronic text file, have been provided to ITT to enable the recommended ECT correction to be made. Use of this recommended correction curve will enable GOES calibration model to be more directly traceable to NIST.

## 7. Concluding Remarks

Satellite remote sensing has the potential to deliver the high accuracy data required to identify and monitor the small signals of climate change in a long time series record. The hallmark for accuracy is pre-launch SI traceable calibrations of sensor performance and post launch validations. The steps to be taken in pre-launch as a best practice are to plan and implement calibration activity from the beginning of the mission, allocate resources as necessary to achieve SI traceability. In this regard, it is best to have calibration experts from NMIs involved from the mission concept through the entire life of the mission in various roles outlined in this paper. Several research and development plans involving NIST are underway to improve accuracy for space- based radiometry for climate data records. Ideally, the celestial bodies such as sun, moon and stars can function as calibration transfer sources if their radiances are absolutely calibrated and made SI traceable. Both the workshops held in recent years for improving accuracy in remote sensing radiometry [1,2] highly recommended this task. Current knowledge of the lunar spectral irradiance is based on the work of Kieffer and Stone [45] who developed the RObotic Lunar Observatory (ROLO) based in Flagstaff, Arizona. They developed a model (ROLO model) to predict the lunar irradiance across numerous spectral bands of interest in the VIS/NIR spectral region. The ROLO model corrects for lunar phase and libration and provides a time series prediction for satellite sensors to use for calibration. The estimated absolute uncertainty in their temporal model predictions is around 3 % (*k* = 1) where as the relative uncertainty is estimated better than 1 % (*k* = 1). A preliminary study at NIST showed that an absolute uncertainty of 1 % (*k* = 1) or less is achievable in calibrating the moon as a transfer standard in VIS/NIR spectral region by using suitable spectral radiometer observations from high mountain tops to avoid atmos phere effects and coupling with radiometer observations from high altitude balloon flights [2]. However, based on the observations of SeaWiFS, MODIS and other satellites, the use of the moon as a stability monitor for the sensor on orbit for the VIS/NIR spectral region is found to be very effective. To do so is a sound practice as the moon is a very stable reflector of sun’s radiation in that spectral region.

There is considerable effort from the Laboratory of Astronomy and Space Physics (LASP) of the University of Colorado, Boulder to calibrate total solar irradiance measurement (TIM) and spectral irradiance measurement (SIM) instrumentation directly with NIST standards to establish SI traceability for their measurements from space. Also NIST has initiated a project to improve the calibration of stars which can be used as transfer standards by working with the astronomy community. In the thermal and long wavelength infrared spectral region, the CLARREO incubator project plans to use fixed point blackbodies and monitor the cavity emissivity while on orbit and is working with NIST to implement and improve the uncertainty budget and build SI traceability.

## Figures and Tables

**Fig. 5:**
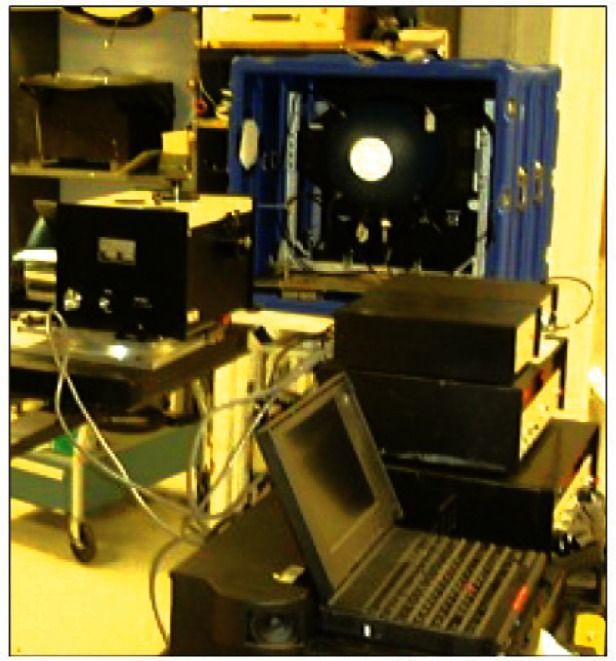
NPR: The NPR and the GSFC scanning monochromator during the EOS intercomparison at NASA’s Ames facility in 1999 [18]. The NPR system is packaged in two shipping containers, the sphere is shock-mounted in the upper blue case, and the electronics in the lower white case.

**Fig. 6a:**
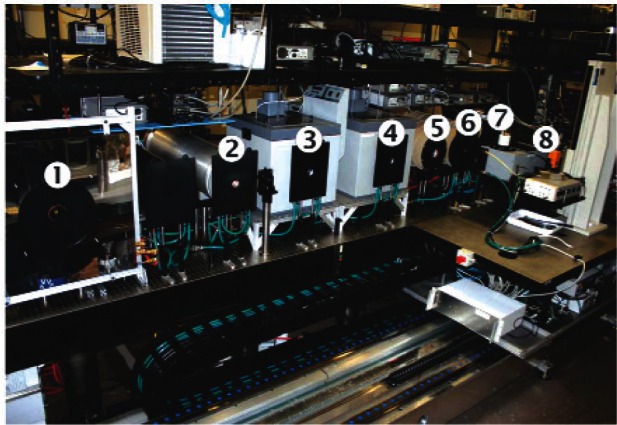
Variable temperature heat pipe Blackbodies (BB) at the AIRI Facility: 1. Controlled Background Plate for Unit Under Test; 2. Ammonia BB (−50 °C to 50 °C), 3. Water Bath BB (15 °C to 75 °C), 4. Water heat pipe BB (60 °C to 250 °C), 5. Cs heat pipe BB (300 °C to 650 °C) and 6. Na heat pipe BB (500 °C to 1100 °C), 7. Spectral comparator (3 μm to 14.8 μm).

**Fig. 6b:**
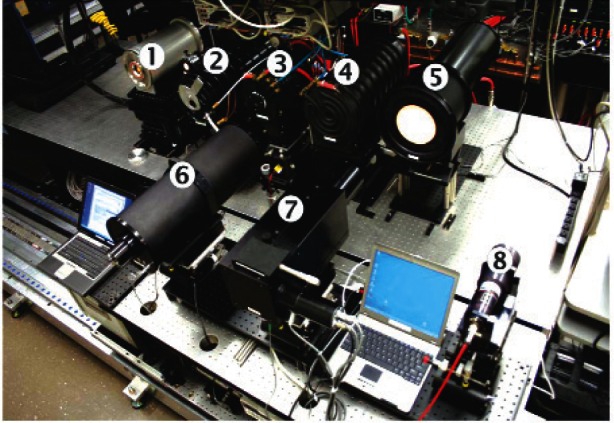
Fixed Point Blackbody (BB) Bench of the AIRI Facility: 1. Ga BB; 2. High T Furnace #1 (Al, AgandAu), 3. Low T Furnace (In, Sn and Zn), 4. High T Furnace #2 (Al and Ag), 5. Out-of-Field Scatter Tool, 6. NIST Transfer Standard Pyrometer RT1550L (150 °C to 1064 °C), 7. NIST Transfer Standard Pyrometer RT900 (600 °C and higher), 8. Transfer Standard Pyrometer TRT (−50 °C to 300 °C).

**Table 1:** Required accuracies and stabilities for climate variable data sets. The column labeled Signal indicates the type of climate signal used to determine the measurement requirements

	Signal	Accuracy	Stability (per decade)

SOLAR IRRADIANCE, EARTH RADIATION BUDGET, AND CLOUD VARIABLES			
Solar irradiance	Forcing	1.5 W / m^2^	0.3 W / m^2^
Surface albedo	Forcing	0.01	0.002
Downward longwave flux: Surface	Feedback	1 W / m^2^	0.2 W / m^2^
Downward shortwave radiation: Surface	Feedback	1 W / m^2^	0.3 W / m^2^
Net solar radiation: Top of atmosphere	Feedback	1 W / m^2^	0.3 W / m^2^
Outgoing longwave radiation: Top of atmosphere	Feedback	1 W / m^2^	0.2 W / m^2^
Cloud base height	Feedback	0.5 km	0.1 km
Cloud cover (Fraction of sky covered)	Feedback	0.01	0.003
Cloud particle size distribution	Feedback	TBD*	TBD*
Cloud effective	Forcing: Water	Water: 10 %	Water: 2 %
particle size	Feedback: Ice	Ice: 20 %	Ice: 4 %

ATMOSPHERIC VARIABLES			

Cloud ice water path	Feedback	25 %	5 %
Cloud liquid water path	Feedback	0.025 mm	0.005 mm
Cloud optical thickness	Feedback	10 %	2 %
Cloud top height	Feedback	150 m	30 m
Cloud top pressure	Feedback	15 hPa	3 hPa
Cloud top temperature	Feedback	1 K / cloud emissivity	0.2 K / cloud emissivity
Spectrally resolved thermal radiance	Forcing / climate change	0.1 K	0.04 K

ATMOSPHERIC VARIABLES			

Temperature			
Troposphere	Climate change	0.5 K	0.04 K
Stratosphere	Climate change	0.5 K	0.08 K
Water-vapor	Climate change	5 %	0.26 %
Ozone			
Total column	Expected trend	3 %	0.2 %
Stratosphere	Expected trend	5 %	0.6 %
Troposphere	Expected trend	10 %	1.0 %
Aerosols			
Optical depth (troposphere/stratosphere)	Forcing	0.01 / 0.01	0.005 / 0.005
Single scatter albedo (troposphere)	Forcing	0.03	0.015
Effective radius (troposphere / stratosphere)	Forcing	greater of 0.1 μm or 10 % of particle size / 0.1 μm	greater of 0.05 μm or 5 % of particle size / 0.05 μm
Precipitation		0.125 mm/h	0.003 mm/h
Carbon dioxide	Forcing/Sources-sinks	0.001 % by volume / 0.001 % by volume	0.00028 % by volume / 0.0001 % by volume

SURFACE VARIABLES			

Ocean color		5 %**	1 %
Sea surface temperature	Climate change	0.1 K	0.04 K
Sea ice area	Forcing	5 %	4 %
Snow cover	Forcing	5 %	4 %
Vegetation	Past trend	3 %	1 %

* To be determined.

** The 5 % accuracy requirement for Ocean color is for the at-satellite component of the water leaving radiance. The accuracy of an ocean color radiometer must be 0.5 % or better to meet this goal.

**Table 2:** Required accuracies and stabilities of satellite instruments to meet requirements of Table 1. The instrument column indicates the type of instrument used to make the measurement

	Instrument	Accuracy	Stability (per decade)
SOLAR IRRADIANCE, EARTH RADIATION BUDGET, AND CLOUD VARIABLES			

Solar irradiance	Radiometer	1.5 W / m^2^	0.3 W / m^2^
Surface albedo	Vis radiometer	5 %	1 %
Downward longwave flux: Surface	IR spectrometer and Vis / IR radiometer	See tropospheric temperature, water-vapor,cloud base height, and cloud cover	See tropospheric temperature, water-vapor, cloud base height, and cloud cover
Downward shortwave radiation: Surface	Broad band solar and Vis/IR radiometer	See net solar radiation: TOA, cloud particle effective size, cloud optical depth, cloud top height, and water-vapor	See net solar radiation: TOA, cloud particle effective size, cloud optical depth, cloud top height, and water-vapor
Net solar radiation: Top of atmosphere	Broad band solar	1 W / m^2^	0.3 W / m^2^
Outgoing longwave radiation: Top of atmosphere	Broad band IR	1 W / m^2^	0.2 W / m^2^
Cloud base height	Vis / IR radiometer	1 K	0.2 K
Cloud cover (Fraction of sky covered)	Vis / IR radiometer	See cloud optical thickness and cloud to temperature	See cloud optical thickness and cloud to temperature
Cloud particle size distribution	Vis / IR radiometer	TBD*	TBD*
Cloud effective particle size	Vis / IR radiometer	3.7 μm: Water, 5 %; Ice, 10 % 1.6 μm: Water, 2.5 %; Ice, 5 %	3.7 μm: Water, 1 %; Ice, 2 % 1.6 μm: Water, 0.5 %; Ice, 1 %
Cloud ice water path	Vis / IR radiometer	TBD*	TBD*
Cloud liquid water path	Microwave and Vis / IR radiometer	Microwave: 0.3 K Vis/IR: see cloud optical thickness and cloud top height	Microwave: 0.1 K Vis/IR: see cloud optical thickness and cloud top height
Cloud optical thickness	Vis radiometer	5 %	1 %
Cloud top height	IR radiometer	1 K	0.2 K
Cloud top pressure	IR radiometer	1 K	0.2 K
Cloud top temperature	IR radiometer	1 K	0.2 K
Spectrally resolved thermal radiance	IR spectroradiometer	0.1 K	0.04 K

ATMOSPHERIC VARIABLES			

Temperature			
Troposphere	MW or IR radiometeer	0.5 K	0.04 K
Stratosphere	MW or IR radiometer	1 K	0.08 K
Water vapor	MW radiometer	1.0 K	0.08 K
	IR radiometer	1.0 K	0.03 K
Ozone			
Total column	UV/VIS spectrometer	2 % (1 independent), 1 % (1 dependent)	0.2 %
Stratosphere	UV/VIS spectrometer	3 %	0.6 %
Troposphere	UV/VIS spectrometer	3 %	0.1 %
Aerosols	VIS polarimeter	Radiometric: 3 % Polarimetric: 0.5 %	Radiometric: 1.5 % Polarimetric: 0.25 %
Precipitation	MW radiometer	1.25 K	0.03 K
Carbon dioxide	IR radiometer	3 %	Forcing: 1 %; Sources/sinks: 0.25 %

SURFACE VARIABLES			

Ocean color	VIS radiometer	5 %	1 %
Sea surface temperature	IR radiometer	0.1 K	0.01 K
	MW radiometer	0.03 K	0.01 K
Sea ice area	VIS radiometer	12 %	10 %
Snow cover	VIS radiometer	12 %	10 %
Vegetation	VIS radiometer	2 %	0.80 %

* To be determined

## Footnotes

2 Certain commercial equipment, instruments, and materials are identified in this paper to foster understanding. Such identification does not imply recommendation or endorsement by the National Institute of Standards and Technology (NIST).
